# Implementation of mobile medical units for naloxone distribution and MOUD linkage in Ohio’s HEALing Communities Study: a community case study

**DOI:** 10.3389/fpubh.2026.1883800

**Published:** 2026-07-29

**Authors:** Sofia Rubi, Tim Ingram, Jennifer L. Brown, Joel Sprunger, Paul Matherny, Michael S. Lyons, T. John Winhusen

**Affiliations:** 1 Department of Psychological Sciences, Purdue University, West Lafayette, IN, United States; 2 Department of Psychiatry & Behavioral Neuroscience, College of Medicine, University of Cincinnati, Cincinnati, OH, United States; 3 Center for Addiction Research, University of Cincinnati, Cincinnati, OH, United States; 4 Department of Internal Medicine, The Ohio State University, Columbus, OH, United States; 5 Department of Emergency Medicine, The Ohio State University, Columbus, OH, United States

**Keywords:** addiction, implementation, mobile medical units, opioid, prevention

## Abstract

Naloxone, an opioid overdose reversal drug, and medication for opioid use disorder have been found to effectively combat opioid-related overdose and death. However, cost and access remain considerable barriers for many individuals at the highest risk of opioid-related overdoses. Similarly, many struggle to access medications for opioid use disorder because of the limited availability of treatment facilities and inadequate transportation options. Within the broader HEALing Communities Study, some Ohio communities elected to use mobile medical units to support delivery of naloxone and medications for opioid use disorder. This community case study describes the implementation experiences reported by Intervention Facilitators in each county on mobile medical unit deployment, including barriers, facilitators, and operational considerations. The observations provide practical insights that may be useful for future MMU implementation efforts.

**Clinical trial registration:**

ClinicalTrials.gov, identifier: NCT04111939.

## 1 Introduction

The opioid epidemic in the United States was declared a public health emergency in 2017 (1) and was further exacerbated by the COVID-19 pandemic (2). Opioid-related overdoses increased by 18% nationwide in the year following the onset of the pandemic (2). Approximately 8.9 million Americans over the age of 12 have reported misusing opioids within the past year, while only an estimated 55.2% of adults with opioid use disorder (OUD) have received treatment (3–5). In 2022, Ohio had the tenth-highest drug overdose death rate in the country, with an estimated death rate of 38 per one hundred thousand people (3, 6).

Naloxone and medication for opioid use disorder (MOUD), including methadone and buprenorphine, reduce the risk of overdose and mortality (7, 8). However, an estimated 28% of people needing OUD treatment have received MOUD in the past year (9–11). Stigma, financial costs, lack of transportation, and limited access represent barriers to providing OUD prevention and treatment services to those in need (8, 10, 12). Treatment barriers disproportionally impact marginalized populations such as individuals with previous legal involvement and low socioeconomic status, people of color, and individuals living with HIV/AIDS, among others (13, 14). Individuals located in rural communities also face unique challenges accessing treatment for OUD. For residents of a rural-dominated state like Ohio, this is especially true, as many live in large regions considered to be ‘substance-use treatment deserts’ lacking treatment options within driving distance (15). One strategy to support underserved populations affected by OUD is to utilize mobile medical units (MMUs) for community-based prevention and treatment. MMUs can connect individuals with resources, medications, and brief health counseling. This approach is an alternative to existing fixed-site programs in outpatient facilities that are often at capacity or inaccessible to vulnerable communities (16). Providing preventative health care and screening services on MMUs identifies patients most at risk for early disability, injury, and disease and helps reduce future healthcare costs while improving quality of life. For instance, a MMU operating for the past 16 years in Massachusetts demonstrated remarkable cost-efficiency in providing primary care services (17). For every dollar invested in its operation, the unit has saved $36 in related healthcare expenses by reducing emergency department visits (17).

MMUs focused on OUD services may also provide additional preventative services, including syringe exchange programs, infectious disease testing, and overdose response training (16). Using MMUs to address OUD, among other substance use disorders, has been evaluated in several states, including New Jersey, Connecticut, and Maryland; these MMUs have enhanced both community outreach to underserved individuals and fostered greater retention in health services (13, 18). Implementing MMUs may also more effectively connect at-risk populations and marginalized communities with mental or behavioral health treatment and broader healthcare services at a higher rate than fixed-site programs (13, 14, 19). Another HEALing Community Study (HCS) stakeholder implemented an MMU in a predominantly urban, racially diverse setting to engage individuals from diverse racial backgrounds and those experiencing homelessness in MOUD and sexual health services (20). Additionally, MMU efforts implemented in Baltimore, Maryland, observed higher rates of both short- and long-term MOUD treatment retention compared to fixed-site programs, and up to 67.9% of individuals prescribed MOUD sustained treatment for two or more visits following initiation (19, 21, 22).

We describe implementation strategies, contextual challenges, facilitators, and operational considerations of MMUs for naloxone distribution within eight Ohio communities, linking individuals to MOUD treatment and other services to mitigate opioid-related overdoses and deaths as a part of the larger HCS study. This community case study can inform future MMU implementation in other communities to address the opioid epidemic.

## 2 Context

HCS was a parallel-group, cluster-randomized, unblinded, wait-list controlled trial of 67 communities in four states—Kentucky (*n* = 16), Massachusetts (*n* = 16), New York (*n* = 16), and Ohio (*n* = 19)—with at least 30% of communities within each state being rural. Communities were the unit of analysis and consisted of counties (*n* = 48) or cities/towns (*n* = 19; 16 in Massachusetts; and 3 in New York). Counties were those with a baseline rate of opioid-related overdose deaths of ≥25 per 100,000 adults (23, 24). The conceptualization of HCS (12, 25), the study protocol (23), the Communities That Heal intervention (26), and the guiding implementation science frameworks have been previously published (27). Briefly, community coalitions were formed or adapted from existing coalitions and, based on a data-informed needs assessment, selected strategies to implement from the Opioid-Overdose Reduction Continuum of Care Approach, a compendium of evidence-based practices (EBPs) to reduce opioid overdose mortality. Communities were required to implement at least one strategy from each of five menu categories of EBPs: (a) overdose education and naloxone distribution (OEND); (b) expanding MOUD treatment availability; (c) expanding linkage to MOUD services; (d) supporting MOUD treatment engagement and retention; and (e) safer opioid prescribing and dispensing practices. MOUD EBP strategies focused specifically on buprenorphine and methadone.

Communities highly impacted by opioid-related overdose fatalities (a rate of ≥25 deaths per 100,000 people) based on 2016 data were recruited to participate. Communities were randomized into Wave 1 or 2 of intervention implementation and were required to implement at least one strategy related to OEND. Community-based interventions were delivered from January 2020 through June 2022 to Wave 1 communities and from July 2022 through December 2023 to Wave 2 communities.

## 3 Detail to understand key programmatic elements and methodology

Study arm allocation was executed using a covariate-constrained randomization procedure performed within each state. Constraint variables included urban/rural classification, baseline opioid overdose death rate, and community population. The protocol (Pro00038088) was approved by Advarra Inc., the HCS single Institutional Review Board, and was granted a Waiver of Consent and a Full Waiver of HIPAA Authorization for secondary data analysis (3/6/2023, MOD00521925). The Data Safety Monitoring Board, chartered by the National Institute on Drug Abuse, was an independent group charged with monitoring the safety of participating communities.

Intervention Design Teams were created to assist in the development of EBP implementation plans and provided ongoing technical facilitation to support EBP implementation. This model allowed for the integration of individual expertise and lived experience through peer-to-peer technical facilitation to support service agency partners’ EBP implementation efforts. Relevant to the current study was the technical facilitation regarding the purchase and use of vehicles as MMUs for OEND and MOUD services implemented across settings. The public health and community-based organizations’ Intervention Design Team oversaw the technical facilitation for the MMUs. Within the Ohio site, each of the 18 participating counties had a full-time staff member, termed an Intervention Facilitator, dedicated to supporting EBP implementation efforts across the county and reporting study progression. Research investigators affiliated with Ohio academic institutions were responsible for disseminating information regarding implementation activities through peer-reviewed publications, conference presentations, and other information. Intervention Facilitators completed an open-ended follow-up survey developed by research investigators regarding their county’s experience implementing EBP’s. Surveys were distributed in January 2024, and Intervention Facilitators were allotted a 30-day window to complete and submit responses. Survey responses were reviewed and categorized by research investigators into themes related to implementation barriers, facilitating factors, lessons learned, and perceived outcomes. These qualitative data provided valuable insight into the real-world implementation process and were used to inform the description of execution experiences, operational considerations, and recommendations for future implementation efforts presented in this manuscript.

## 4 Results

Across Waves 1 and 2 of the HCS, eight Ohio counties (three urban and five rural) opted to implement EBPs using MMUs. Seven of these eight counties utilized HCS funds to purchase seven new vehicles. Two counties already had MMUs in operation and expanded EBP strategies on two existing units with HCS funds. In total, nine MMUs were involved: seven newly purchased units for the HCS study and two existing units supported by HCS funds. The Ohio counties that purchased MMUs ranged from small rural communities to larger urban centers with population sizes ranging from 38,000 to 1.2 million and were in all geographic regions of Ohio (see Table 1). In Wave 1, five counties implemented a single MMU, and one county implemented two; two counties in Wave 2 implemented a single MMU, and one county implemented an EBP strategy on an existing MMU. Counties used local knowledge and available information from emergency responders, health departments, and community partners to inform MMU deployment during Waves 1 and 2. Areas perceived to be experiencing elevated overdose activity were deemed as high-risk ‘hotspots’ and were prioritized for MMU service delivery. All Intervention Facilitators completed the follow-up survey on behalf of their assigned county.

**Table 1:** Characteristics of Ohio HCS counties that implemented mobile medical units. (Source: US Census 2020).

Implementation site characteristics	County 1	County 2	County 3^*^	County 4	County 5	County 6	County 7	County 8^*^
Urban/rural	Rural	Urban	Rural	Urban	Rural	Rural	Urban	Rural
Population	40,000+	400,000+	70,000	1,000,000+	60,000+	90,000+	350,000+	60,000+
Evidenced-based practice	OEND, MOUD linkage	OEND	OEND, MOUD linkage	OEND, MOUD linkage	OEND	OEND, MOUD linkage	OEND, MOUD linkage	OEND, MOUD linkage
Mobile medical unit status	Purchased	Purchased	1 Purchased & 1 Supported	Purchased	Purchased	Purchased	Purchased	Supported
Number of mobile medical units	1	1	2	1	1	1	1	1

These data originate from the 2020 United States Census. OEND, overdose education and naloxone distribution; MOUD, medications for opioid use disorder; MOUD linkage, a strategy in which individuals are assisted with establishing MOUD treatment; Purchased, mobile medical unit was purchased with assistance from HCS; Supported, HCS supported an existing mobile medical unit in the county; ^*^Counties with mobile medical units in operation before the start of HCS.

### 4.1 MMU services and implementation

All nine MMUs incorporated OEND into their services. As a best practice, overdose education was based on a curriculum developed by HCS experts and was standardized across MMUs. Staff and clients received instruction that covered recognizing overdose symptoms, understanding the risk associated with substance adulterants such as synthetic opioids, and highlighting the importance of carrying naloxone. HCS also utilized handouts, group instructions, and social media, including Facebook and Twitter/X, to inform community members about the signs of opioid overdose and the proper use of naloxone. Naloxone was distributed in “kits” that included two doses of naloxone nasal spray, detailed instructions for naloxone administration, personal protective equipment, OUD treatment contact information, and disposal bags for pills. Additionally, MMU staff provided verbal instructions on the use of naloxone with a handout outlining steps on how to respond to an opioid overdose (i.e., call 9-1-1, administer the naloxone, and administer CPR). These kits were given to individuals who requested naloxone for themselves or others.

Five of the seven counties that aimed to utilize the MMU implemented this MOUD linkage to treatment strategy. Contact information to nearby treatment facilities and health care professionals was provided to all individuals who demonstrated interest or requested a naloxone kit. In one rural county, a social worker was assigned to the MMU to initiate clinical assessments and treatment planning, thus expediting MOUD accessibility. Additionally, staff members contacted nearby treatment facilities to ensure appointment availability. In some cases, clients were able to obtain appointments as early as the same day. In an urban county, transportation services to and from treatment facilities were also offered to clients through transportation vouchers.

Two rural counties sought to increase access to MOUD via telehealth via the MMU; however, one county ultimately did not adopt this service. The primary reasons for this were a lack of demand and the requirement of a completed clinical assessment and treatment plan for MOUD prior to the telehealth appointment. Instead, the county that did not adopt telehealth implemented a program that utilized a nurse practitioner to initiate MOUD via telehealth services facilitated by the MMU.

## 5 Discussion

### 5.1 Stigma and skepticism

Several county coalitions initially questioned the efficacy and need for MMU services providing linkage to MOUD treatment and OEND within their community. Overdose education encompasses broader content related to overdose prevention and response; it also includes education regarding opioid use, opioid overdose risk factors, overdose recognition, and naloxone administration. Difficulty engaging law enforcement and EMS staff in OEND strategies further established misconceptions about the necessity of these initiatives. Outreach efforts were hindered by the public stigma surrounding MMU services, complicating the organization of events and advertising aimed toward engaging community members. Stakeholders speculated that community member resistance influenced public officials’ willingness to actively promote health care provided by MMUs. In some rural communities, members showed limited interest in MMU services and utilization of MOUD treatment referrals. In other counties, previously designated sites for MMU services were barred from further use due to negative perceptions of the services they provided.

Additional education was required to combat stigma and assure residents of the value of these services to their communities. One rural county leveraged HCS funding to provide formal opioid training and education that included testimonials from residents who had lost a loved one to overdose. Additionally, leaders representing EMS and the medical community publicly endorsed these EBPs as a viable solution for addressing the opioid epidemic. The training also included addiction specialists to offer empirical information about addiction and address the stigma and misconceptions surrounding the topic. Local advocates also played a critical role in challenging and neutralizing the prevailing stigma. Such advocates included leaders with significant social capital in the community, including physicians, business owners, parents who had lost a child to overdose, faith-based leaders, and elected officials who recognized the significance of the MMU.

Peer-to-peer consultation across Ohio coalitions using MMUs proved helpful to those seeking information on services offered, sustainability costs, staffing levels, marketing, and prime MMU locations. HCS staff facilitated these consultation meetings with coalition members and peer organizations, both virtually and in person, to provide insight on MMUs already purchased and operational in other communities. Occasionally, coalition members visited other counties to observe the MMU firsthand.

The COVID-19 pandemic further highlighted the need for MMU services by disrupting access to traditional healthcare and substance use treatment (2). Public health measures, including social distancing, combined with significant strain on healthcare systems, created an urgent need for innovative approaches to delivering essential services directly to communities (29). At the same time, the pandemic was associated with increases in opioid use and opioid-related overdose deaths, further exacerbating existing health disparities (2). This emerging need also increased the acceptability of MMU services across participating counties, particularly in rural communities where access to healthcare and substance use treatment was already limited (15). As traditional models of care became more difficult to access during the pandemic, MMUs were increasingly recognized by Intervention Facilitators and community members as a practical and effective approach for bringing essential services directly to underserved populations.

### 5.2 Community leadership and engagement

Each county had a local coalition dedicated to addressing the opioid epidemic that led to the EBP selection process in HCS. Typically, coalitions consisted of approximately 24 individuals ranging from 12 to 46 members. Coalition membership included representatives from elected officials, academia, the coroner’s office, law enforcement, mental health, public health, healthcare, emergency medical services, faith-based organizations, and non-governmental community-based organizations. The coalition structure varied from an executive committee to several committees and subcommittees. Some of the coalitions were already supporting opioid overdose intervention efforts, including the distribution of naloxone and linking individuals with OUD to MOUD treatment services. These coalitions collaborated on the MMU services most appropriate for their communities. Planning prior to the acquisition of the MMU took around 6 to 12 months to determine ownership, staffing and scheduling, storage, and maintenance responsibilities. HCS Intervention Facilitators regularly met with coalitions to build relationships and provide expertise. This was crucial for cultivating trust with local coalitions to progress MMU acquisition and implementation. Early implementation required ongoing operational troubleshooting as MMUs were integrated into existing community workflow, particularly related to aligning MMU operations with local service infrastructure and community expectations. Strong affiliations between the local health department and community partners, other governmental agencies, and businesses aided in identifying secure locations for the MMU to execute its services and outreach events. HCS collaborations led to the use of data-informed approaches to improve implementation strategies by identifying high-risk areas. Targeting these areas allowed MMUs to direct health services to underserved and under-resourced regions of participating counties. In one example, a member of a rural Ohio county recalled positive outcomes associated with their county’s MMU’s, such as linking individuals to mental health and infectious disease services that would not have been possible otherwise.

Organizational partnerships were also important in the acceptance and planning of evidence-based services via MMUs. Studies have shown that collaborating with organizations helps build trust and credibility, especially among at-risk populations (30). Key organizations include local public health departments, mental health agencies, emergency medical services, law enforcement, and other health care and behavioral health service agencies. Many of the counties had one key individual or organization in a leadership position that championed acquiring the MMU for their respective county. Outreach efforts to enhance service utilization varied depending on the characteristics of the community. Most of the counties used social media and existing client networks to market the services offered by the MMUs. One used billboard advertising, while urban counties enlisted partnerships with local media, including newspapers and TV stations, to showcase the services offered and the MMUs in operation. Newspaper articles and agency newsletters were the primary ways the MMUs were spotlighted. Several counties used a texting service called *Textedly* to notify clients of the dates and locations of the MMUs, providing a flexible and direct communication method with beneficiaries.

MMUs made additional initiatives possible, including additional overdose prevention education and expanded county overdose monitoring efforts. Additionally, coalition meetings in some counties have since been expanded to include partnering organizations and stakeholders to strategize future collaborations and to develop sustainability plans to preserve MMU services following the completion of HCS. Several counties described exploring pathways for maintaining MMU services, including leveraging partnerships with local health departments and community organizations. Collectively, these experiences suggest an implementation trajectory characterized by early operational challenges, followed by progressive adaptation and increasing integration of MMU services within local systems.

### 5.3 Limitations

Several limitations should be considered when interpreting these findings. Counties either self-selected or coalition-selected MMUs as an evidence-based practice within HCS, and those that adopted MMUs may have differed from non-adopting counties in leadership capacity, public health infrastructure, harm reduction resources, or political support. Additionally, the funding and technical assistance provided through HCS may not be readily replicable in routine public health settings. County anonymization, while necessary for confidentiality, also limits contextual interpretation of the findings. It should be noted that implementation occurred during the COVID-19 pandemic, which may have impacted service delivery, utilizing patterns, overdose trends, and telehealth adoption. Implementation experiences described may also have been influenced by concurrent policy changes and shifts in illicit drug supply. Future studies should consider incorporating patient and service-level outcome data to better evaluate the impact of MMU-based services on overdose-related outcomes.

## Data Availability

The original contributions presented in the study are included in the article/supplementary material, further inquiries can be directed to the corresponding author.
